# Prosodic influence in face emotion perception: evidence from functional near-infrared spectroscopy

**DOI:** 10.1038/s41598-020-71266-6

**Published:** 2020-09-01

**Authors:** Katherine M. Becker, Donald C. Rojas

**Affiliations:** Department of Psychology, Colorado State University, Campus Delivery 1876, Fort Collins, 80523 USA

**Keywords:** Emotion, Neurophysiology, Human behaviour

## Abstract

Emotion is communicated via the integration of concurrently presented information from multiple information channels, such as voice, face, gesture and touch. This study investigated the neural and perceptual correlates of emotion perception as influenced by facial and vocal information by measuring changes in oxygenated hemoglobin (HbO) using functional near-infrared spectroscopy (fNIRS) and acquiring psychometrics. HbO activity was recorded from 103 channels while participants ($\hbox {n} = 39$, $\hbox {age} = 20.37\ \hbox {years}$) were presented with vocalizations produced in either a happy, angry or neutral prosody. Voices were presented alone or paired with an emotional face and compared with a face-only condition. Behavioral results indicated that when voices were paired with faces, a bias in the direction of the emotion of the voice was present. Subjects’ responses also showed greater variance and longer reaction times when responding to the bimodal conditions when compared to the face-only condition. While both the happy and angry prosody conditions exhibited right lateralized increases in HbO compared to the neutral condition, these activations were segregated into posterior-anterior subdivisions by emotion. Specific emotional prosodies may therefore differentially influence emotion perception, with happy voices exhibiting posterior activity in receptive emotion areas and angry voices displaying activity in anterior expressive emotion areas.

## Introduction

Affective cues communicated by the face and voice of another individual are integrated by the brain to form whole percepts of emotion^1^. The role of emotional facial expressions has been further refined, and it has been suggested that all facial expressions can be categorized as belonging to one of six basic emotions (happiness, sadness, anger, surprise, fear, and disgust)^2,3^, each of which is distinguished by the unique engagement of different sets of facial muscles^4^. Voices transmit auditory analogs of the six basic emotions by altering the rhythm, intensity, and intonation of a speaker’s voice (i.e., prosody)^5–8^. Additionally, vocal and facial expressions of emotion are inherently linked as affective vocalizations normally coincide with and are produced by the coordinated actions of multiple vocal and facial muscles^5,7^. While these mechanisms may be innately linked at the physiological level, each modality appears to provide sufficient affective information to be accurately and independently recognized and identified^2,5,7^. These findings indicate that emotion appears to be relatively well conserved across sensory modalities, allowing for multiple representations of the same internal state.

Faces are processed via a distributed network of brain regions spanning cortical and subcortical occipitotemporal areas, which differentially contribute to face perception. These areas include the fusiform face area, amygdala, posterior-temporal cortices, and ventral parietal cortices, as well as some somatosensory areas^9^. Findings from functional magnetic resonance imaging (fMRI) and electroencephalography (EEG) have shown that the posterior superior temporal sulcus (pSTS) exhibits increased activity when viewing lip, eye, and cheek movements^10–15^. These dynamic facial features are essential to all aspects of speech production as findings from EEG and fMRI have shown that the pSTS exhibits increased activity when viewing dynamic faces, listening to affective voices^11,16,17^, and during silent lip reading^18^. These findings suggest that the pSTS may possess a holistic representation of social communication, which incorporates both the changeable aspects of faces and their concomitant vocalizations.

Voices supplement visual facial information by providing an auditory correlate of an expressed emotion^1^. Affective language is differentially represented in the right hemisphere in a functional-anatomical organization that parallels that shown for propositional language in the left hemisphere^19,20^. Findings from lesion studies in aprosodia patients have shown that damage to structural homologues of Wernicke’s and Broca’s area in the right hemisphere produce deficits in comprehending and reproducing prosody similar to those seen in patients with receptive and expressive aphasias^19,20^. fMRI studies have shown that these areas may possess distinct representations of emotion as areas of the pSTS and superior temporal gyrus (STG) exhibit unique, emotion-specific patterns of activation when presented with nonverbal affective voices^21–24^. EEG recordings have shown that affective vocalizations are processed in the STS with increasingly complex processing occurring as activity moves from posterior to anterior temporal areas where emotional information is integrated and then transferred to frontal brain areas for higher-order cognitive processing^25^. The pSTS appears to play an essential role in audiovisual speech perception by integrating the emotional content of a speaker’s voice with their concomitant facial expression to aid in speaker identification and affect recognition^26^.

Together, these findings indicate that socially relevant audiovisual stimuli converge and engage partially overlapping regions in the pSTS/G^1,27–29^. This functional convergence underscores the multidimensional nature of affect perception, in that, regardless of the physical medium, presentation of a face, voice, or body movement impresses upon the observer the same perceptual experience^30,31^. The neural correlate of this supramodal representation appears to occur in the pSTS, wherein patterns of activity change between emotions, but not between different sensory modalities conveying an emotion^27,32,33^. This emotional specificity can even detect incongruencies between affective vocal and facial input, as the pSTS exhibits increased activity to mismatched happy and angry stimuli^34^. Additionally, simultaneous presentation of affective voices and faces elicits activity in areas identical to those when voices and faces were presented separately^32^. This overlap may represent the common engagement of several neural structures, which support the perception, experience, and expression of emotion^35^.

Positioned slightly superior to the pSTS, located at the posterior end of the Sylvian fissure sits the temporoparietal junction (TPJ). TPJ appears to act as a supramodal association area for visual and auditory information related to social cognition and has been implicated in several prosocial cognitive functions: language, episodic memory encoding, reorienting attention, mentalizing, and altruism^36^. The convergence of these socially relevant cognitive processes may reflect the integration of sensory and conceptual information that occurs in posterior brain areas that become engaged when attempting to connect with, respond to, or take the perspective of another person^37,38^. These processes promote positively valenced affiliative behaviors which foster emotional mimicry, empathy, and relationship formation^39^. Moreover, this area is essential to receptive paralinguistic vocal communication as the aprosodia literature has shown that damage to temporoparietal areas may lead to deficits in comprehending affective prosody^19,20^. This impairment appears to obscure emotion perception by interfering with an individual’s ability to properly interpret the prosody of another person’s voice, potentially resulting in communication deficits and difficulties in maintaining relationships^40^. Thus, damage to these brain regions may impair crucial aspects of psychosocial functioning that are involved in connecting with, listening to and understanding another individual’s emotional state.

Conversely, damage to right frontal areas has been shown to lead to deficits in expressive prosody, wherein an individual is unable to reproduce examples of affective prosody^19^. These regions may hold special significance in the interpretation and expression of negatively valenced emotions as right frontal areas have been associated with responding to and understanding others’ anger^41,42^. Aprosodia patients have been shown to exhibit deficits in expressing extreme emotional states and this may be dissociable from the expression of less extreme emotions, which may be localized to different brain areas^40^. Moreover, these areas appear to be crucial to expressing anger, as patients with right frontal lesions struggle in mimicking angry, but not happy, faces^42^. These findings indicate that anterior and posterior brain areas in the right hemisphere may be differentially recruited due to the inherent communicative differences exhibited during expressions of an angry or happy emotion^43,44^, with each evoking distinctly different behavioral responses^45,46^.

The supramodal representation of emotion may also be evidenced by the ability of affective faces and voices to bias emotion perception, as studies have indicated that both voices^30,31,47^ and faces^30^ can bias the emotional categorization of the other modality. This perceptual bias was revealed by measuring the point of subjective equality (PSE) and just noticeable difference (JND) values for subjects’ emotional ratings of linearly morphed images of emotionally ambiguous faces created from one happy image and one sad image, which were paired with either a happy or sad voice^31^. These measures were used to calculate the perceptual shift in subjects’ responses and the variance of those responses, respectively. Results showed that faces were judged to be “happier” or “sadder” depending on the prosody of the speaker’s voice^31^. This reciprocal interaction suggests that visual and auditory inputs may be instantaneously combined and processed in overlapping brain areas to affect emotion perception. However, the neural correlates of this perceptual bias are not yet clear.

While the number of multimodal imaging studies investigating the interaction of these channels has steadily increased^48^, most of the findings within the emotion perception literature have only used images of static faces (e.g.,^31,49^), with relatively fewer studies incorporating affective vocalizations^7,31,50^. This disparity may be related to the assumed similarity between the processing of emotional visual and auditory stimuli^51^, with faces serving as a prototype of how affective expressions in other modalities should be processed^1,51^. Such speculation undermines the complex conceptual and paralinguistic information carried by a speaker’s voice^52^. Controlling the linguistic content of vocal stimuli is of critical importance in the interpretation of multimodal studies as verbal stimuli may unintentionally activate language areas, which are unrelated to the emotion of interest^21^. Vocalizations containing verbal or semantic information exhibit patterns of neural activity, which are different than those elicited by nonverbal prosodic voices^21,25,53^. This division is striking as paralinguistic, nonverbal vocalizations selectively elicit activity in the temporal lobe of the right hemisphere, while verbal utterances are associated with bilateral activity in language areas^1,25^. Additionally, a failure to assess voice and face stimuli both simultaneously and independently makes some multimodal findings more difficult to interpret, as there is no separate account of how each modality was differentially processed when presented alone or together^52^. Further elucidating the neural substrates of emotion perception will require the use of multimodal stimuli, which encapsulate both the visual and auditory components of affect perception.

The current study elected to use affective multimodal stimuli to investigate the neural activity underlying multimodal integration during emotion perception using functional neuroimaging and psychophysical measures. Functional near-infrared spectroscopy (fNIRS) is well-suited to study the cortical activity associated with emotion perception, as it allows for the measurement of both oxygenated and deoxygenated blood flow, it does not require immobilization, is resilient to movement, and is a reliable measure of neural activity^54,55^. Additionally, fNIRS measures the hemodynamic response at a higher temporal resolution than fMRI, which makes it well-suited to capture the temporal complexities of emotion perception. While fNIRS has been used in a variety of cognitive tasks, it has been used less frequently in tasks investigating emotion, with very few investigating the integration of emotional audiovisual stimuli^54–57^. Importantly, fNIRS data acquisition is virtually silent, which gives it a significant advantage in sound sensitive studies examining auditory processing as fMRI scanners are inherently limited by the loud noise generated during scans. This extraneous noise may disrupt normal stimulus processing and introduce extraneous stimulus-locked artifacts, which may further complicate later data analysis. Moreover, this study adds to the current literature by presenting data from high density recordings in healthy, awake adults.

We aimed at examining the influence of non-verbal prosodic vocal stimuli on emotion perception of simultaneously presented faces. Functional near-infrared spectroscopy (fNIRS) was used to quantify changes in oxygenated-hemoglobin (HbO) while subjects were presented with affective voices, faces, and voices and faces presented together. We hypothesized that the prosody of the simultaneously presented voice would bias participants’ perception of the stimuli and this bias would be evidenced by an anterior-posterior shift in neural activity between prosody conditions in the right hemisphere, with the angry prosody condition showing an anterior distribution of activity. For the region-of-interest analysis we predicted that activation in bilateral scalp areas consistent with the TPJ would vary with the stimulus type and prosody of the speaker’s voice, with the combined face and voice (F + V) conditions exhibiting greater HbO activity than the face only or voice only conditions. From behavioral data on the same task, we also predicted that the presence of prosodic vocal information would bias the emotion perception in the direction of the vocal emotion in simultaneously presented stimuli in the F + V condition.

## Results

### Behavioral data

**Figure 1:**
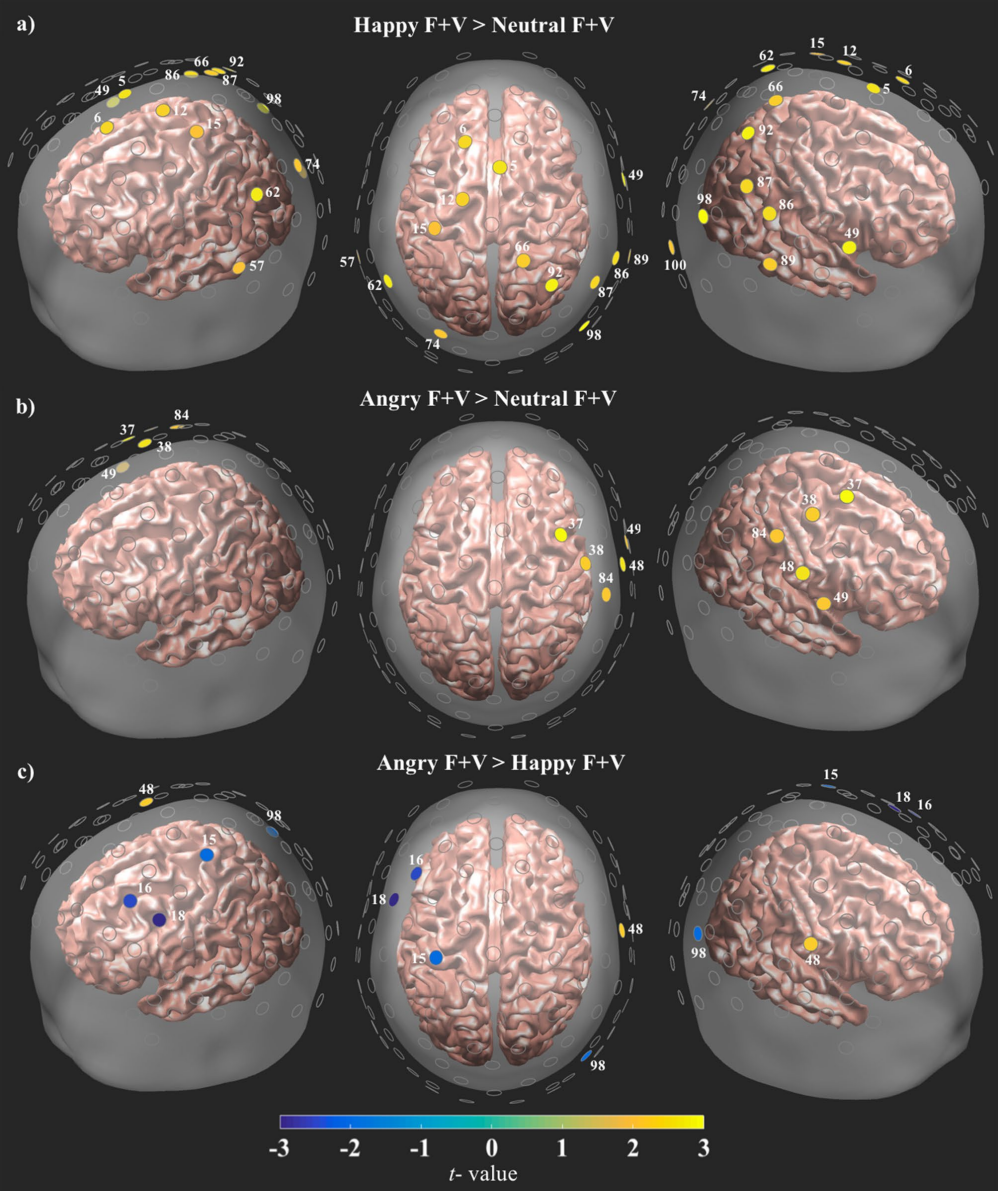
(**a**) and (**b**) show mean point of subjective equality (PSE) and just noticeable difference (JND) values for all face + voice (F + V) and face only conditions, respectively. (**c**) Mean reaction times across the face continuum for each condition. Line colors correspond to those used in the JND and PSE graphs, blue is happy F + V, red is angry F + V, gray is neutral F + V, and green is the face only condition. Significance values are indicated by, $$p < .000 =***$$, $$p < .005 = **$$, $$p < .05 = *$$.

To analyze the hypothesized bias effects, data were fit using a logistic function to calculate point of subjective equality (PSE) and just noticeable difference (JND) values, which were analyzed using two identical one-way repeated measures analysis of variance (ANOVA), with condition as the within subject factor. PSE and JND values for each condition are located are reported in Supplementary Table 8S. There was a statistically significant difference in PSE values between conditions $\hbox {F}(3{,}115) = 8.48$, $p =.0000$ with the Happy prosody condition having a significantly different PSE ($3.78 \pm .946$) than both the Angry ($4.87 \pm 1.14$, $p = .0000$) and Neutral ($4.70 \pm .966$, $p =.0003$) conditions. The PSE for the face only condition ($4.15 \pm .628$) was also significantly different than the Neutral ($p = .0281$) and Angry ($p = .0035$) conditions, but not the Happy prosody condition ($p = .1324$), see Fig. 1a. The ANOVA for JND revealed a statistically significant difference in JND values between conditions $\hbox {F}(3,115) =13.39$, $p = .0000$. The face only condition exhibited a significantly larger JND ($3.32 \pm .939$) than all prosody conditions (Happy, $1.77 \pm .689$, $p = .0000$; Angry, $2.04 \pm 1.20$, $p = .0000$; Neutral, $2.16 \pm 1.20$, $p = .0000$). The JND for the Happy prosody condition was not significantly different from the Angry ($p = .3131$) or Neutral ($p = .1494$) prosody conditions, which also did not significantly differ from one another ($p = .6557$), see Fig. 1b. Subject reaction times were analyzed using an ANOVA with the same within subjects factors and levels, which revealed a significant main effect for face step $\hbox {F}(6,162) = 11.65$, $p = .0000$, condition $\hbox {F}(3,81) = 20.01$, $p = .0000$, and a significant interaction between face step and condition $\hbox {F}(18,486) =3.15$, $p = .0000$. Pairwise comparisons revealed significantly faster reaction times for the face only condition when compared to all prosody conditions (Happy, $760.62 \pm -51.08$, $p = .0000$; Angry, $750.106 \pm -40.57$, $p = .0001$; Neutral, $753.73 \pm -44.20$, $p = .0000$). There were no significant differences in reaction times between the prosody conditions (see Fig. 1c).

### NIRS results

Three optodes (two emitters and one detector) were eliminated from all scans as they failed quality control in more than half of the subjects’ datasets. A gain check was used to assess individual channel quality channels with gains higher than 6 but otherwise passing quality controls were interpolated to form 103 channels to ensure that all subjects shared a common set of channels for comparisons.

#### Face and voice contrasts

**Figure 2:**
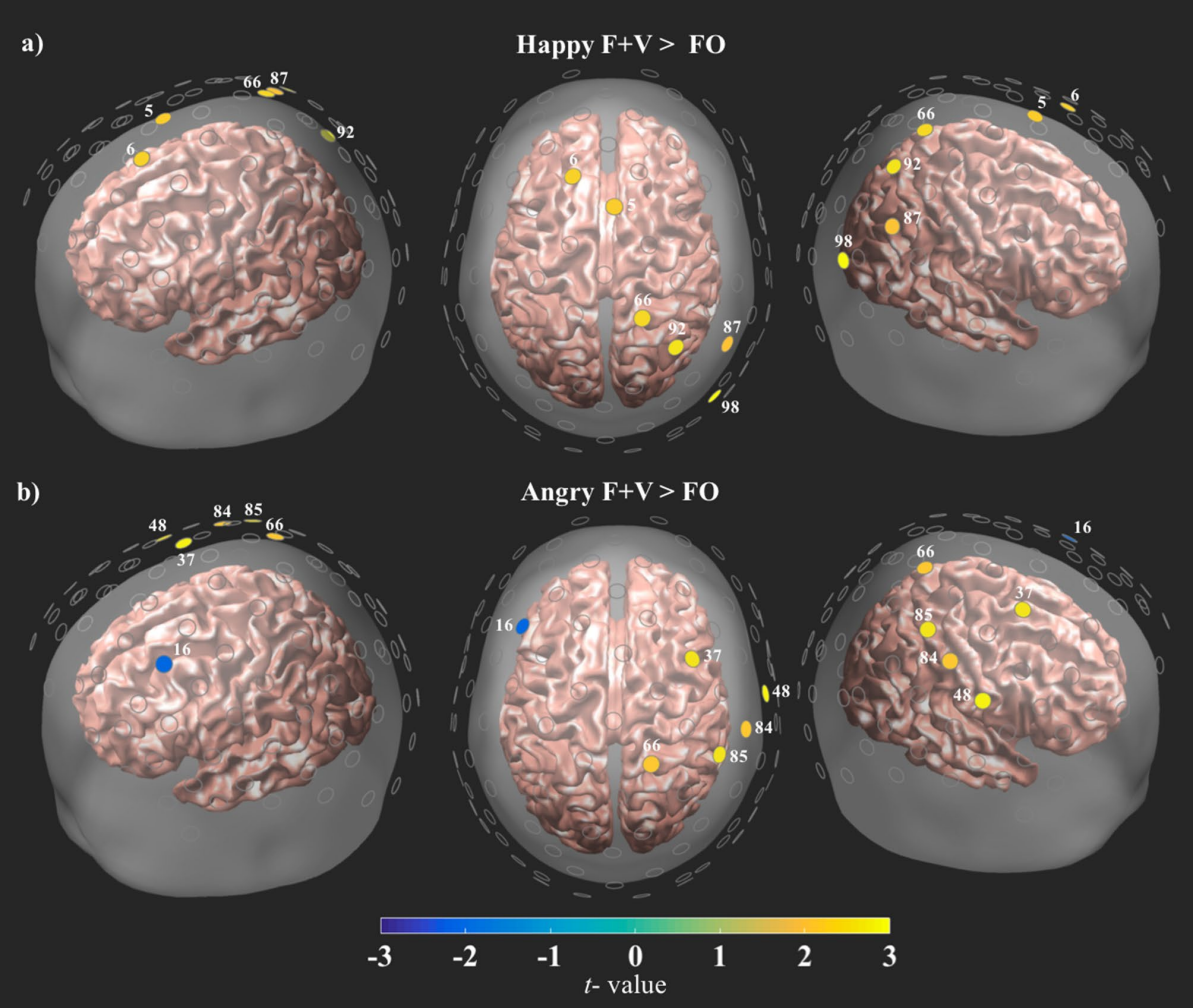
Results of all the bimodal F + V condition contrasts. Channel locations in Fig. 1 and all other results figures are shown overlaid onto a semi-transparent scalp and standard single subject MRI template from SPM 12 for ease of spatial reference. All channels are plotted as gray circles. Channels with significant differences in HbO are shown in yellow or blue, with the associated channel number shown in white. The magnitude and direction of t-values is represented by the color bar shown on the bottom of the figure. All channels survived FDR correction at $\hbox {q} = .05$.

**Figure 3:**
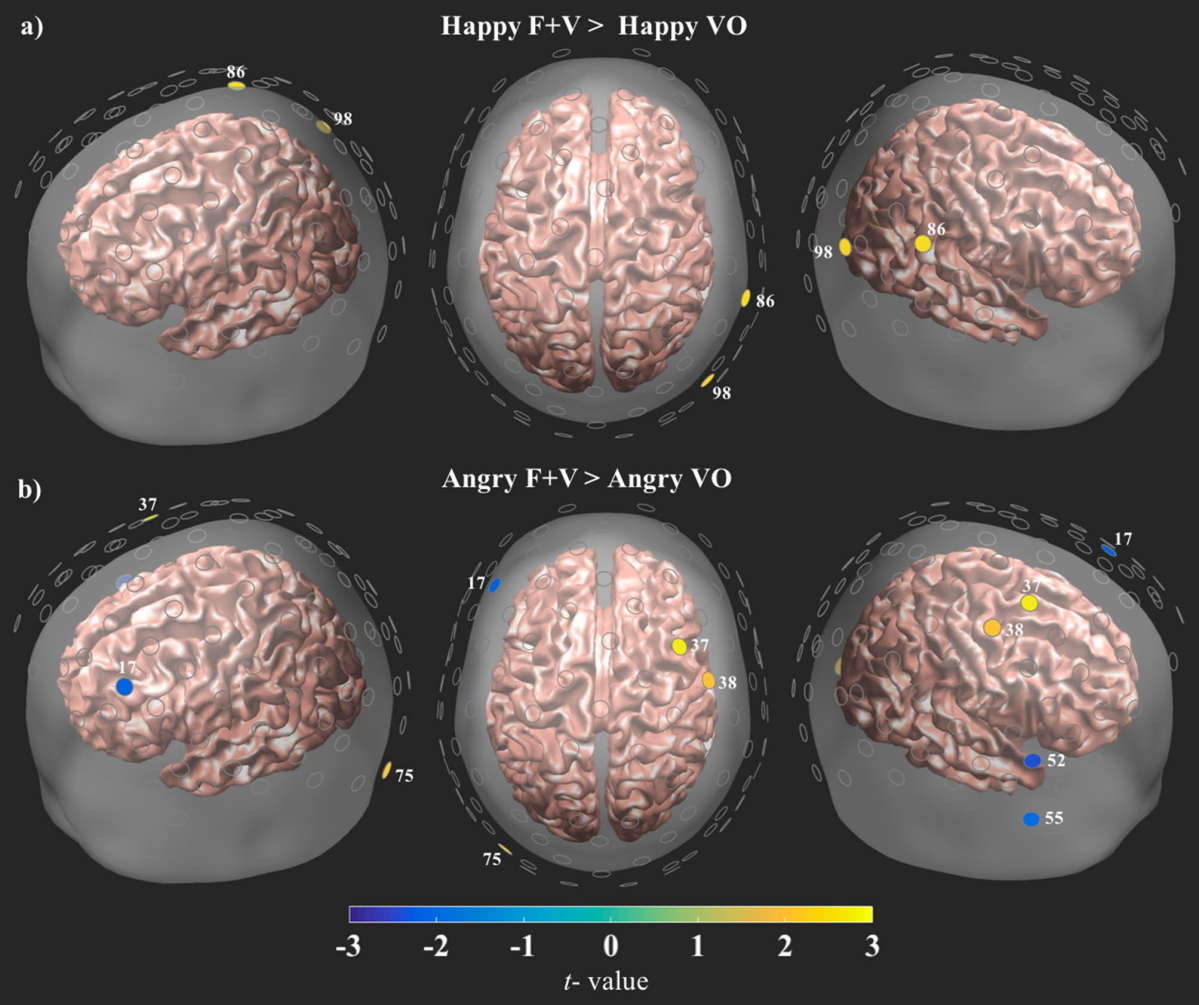
Results of all bimodal F + V and face only (FO) contrasts. All channels are plotted as gray circles. Channels with significant differences in HbO are shown in yellow or blue, with the associated channel number shown in white. The magnitude and direction of the t-values is represented by the color bar shown on the bottom of the figure. All channels survived FDR correction at $\hbox {q} = .05$.

**Figure 4:**
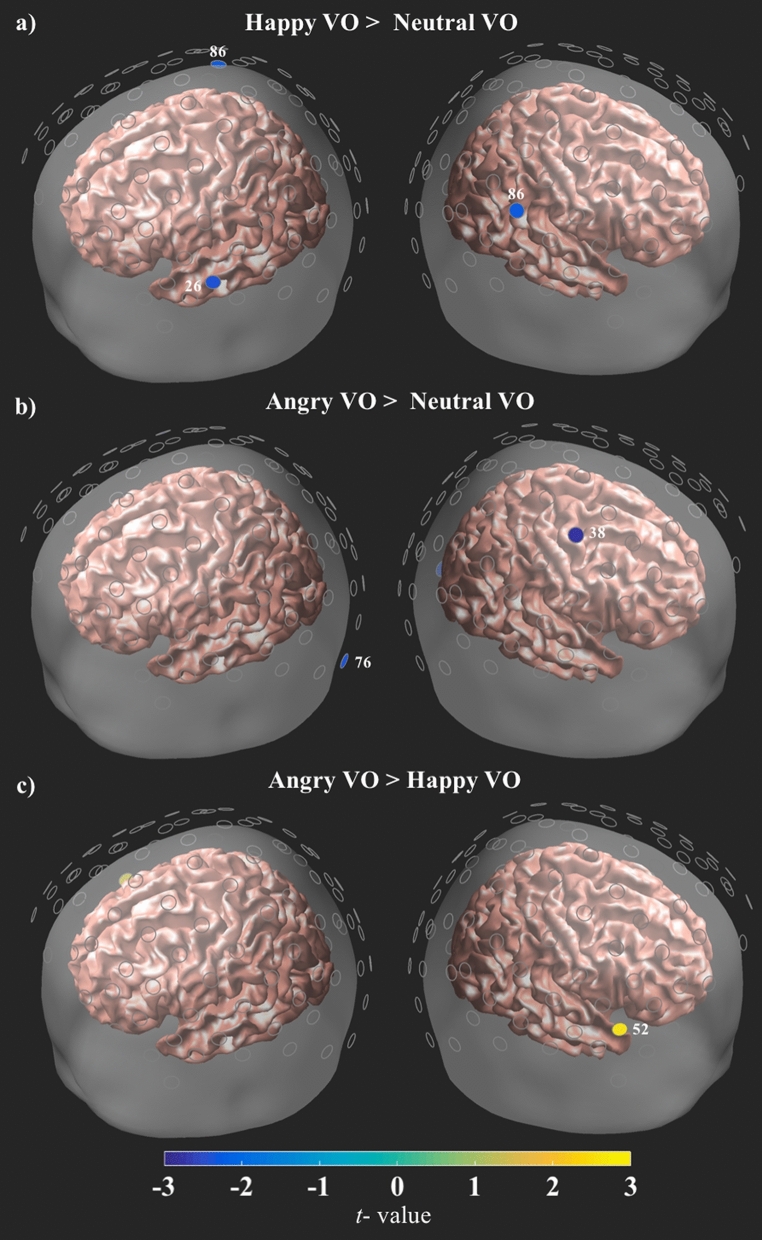
Contrasts for the angry and happy F + V > voice only (VO) conditions. All channels are plotted as gray circles. Channels with significant differences in HbO are shown in yellow or blue, with the associated channel number shown in white. The magnitude and direction of the t-values is represented by the color bar shown on the bottom of the figure. All channels survived FDR correction at $\hbox {q} = .05$.

While both the Angry and Happy F + V conditions exhibited greater concentrations of HbO when compared with the Neutral F + V condition, the resulting patterns of activity were distinctly different (Fig. 2, for full description of the results see Supplementary Table 1S). The Angry-Neutral F + V contrast revealed a pattern of increased HbO that was primarily restricted to motor areas in the right hemisphere (Fig. 2b). Such activations spanned prefrontal areas, with increased HbO occurring in superior and inferior aspects of the precentral gyrus. A handful of channels also exhibited significantly greater activity when compared to the Neutral F + V condition: supramarginal gyrus, middle superior temporal gyrus (STG), inferior postcentral gyrus, and the inferior occipital gyrus (IOG). The Happy-Neutral F + V contrast exhibited a strikingly different pattern of activity, with increased levels of HbO occurring bilaterally (Fig. 2a). Activations in the right hemisphere spanned posterior dorsal areas, which included superior parietal lobules (SPL), superior, middle, and inferior occipital gyri. Bilateral activations appeared in left and right temporoparietal junctions (TPJ), as well as posterior and middle sections of the STG. In the left hemisphere activations appeared in prefrontal and precentral gyrus areas, superior frontal gyrus (SFG), and the TPJ. When compared against one another, the Angry F + V condition exhibited greater HbO in right inferior precentral gyrus than the Happy F + V condition (Fig. 2c). Interestingly, the Happy F + V condition exhibited greater HbO in the right superior occipital gyrus, left dorsolateral prefrontal areas, and superior postcentral gyrus than the Angry F + V condition. When compared to the FO condition, both the Angry and Happy F + V conditions exhibited activity in the right hemisphere, with a similar anterior-posterior division as the previous F + V − F + V contrasts (Fig. 3, see Supplementary Table 2S for more detailed results). Activity included but was not limited to the TPJ for both contrasts. The Happy F + V condition exhibited increased HbO activity in posterior occipital and dorsal association areas (Fig. 3a). Activity for the Angry F + V condition was primarily localized to motor areas (Fig. 3b). Additionally, while greatly reduced, a similar increase in right lateralized anterior-posterior HbO activity appeared when both the Angry and Happy F + V conditions were compared with their respective VO conditions (Fig. 4, see Supplementary Table 3S). Happy F + V activity appeared in right TPJ and posterior occipital areas. The Angry F + V exhibited increased HbO in frontal motor areas but showed decreased HbO in anterior portions of the right temporal lobe (Fig. 4b).

#### Voice only contrasts

**Figure 5:**
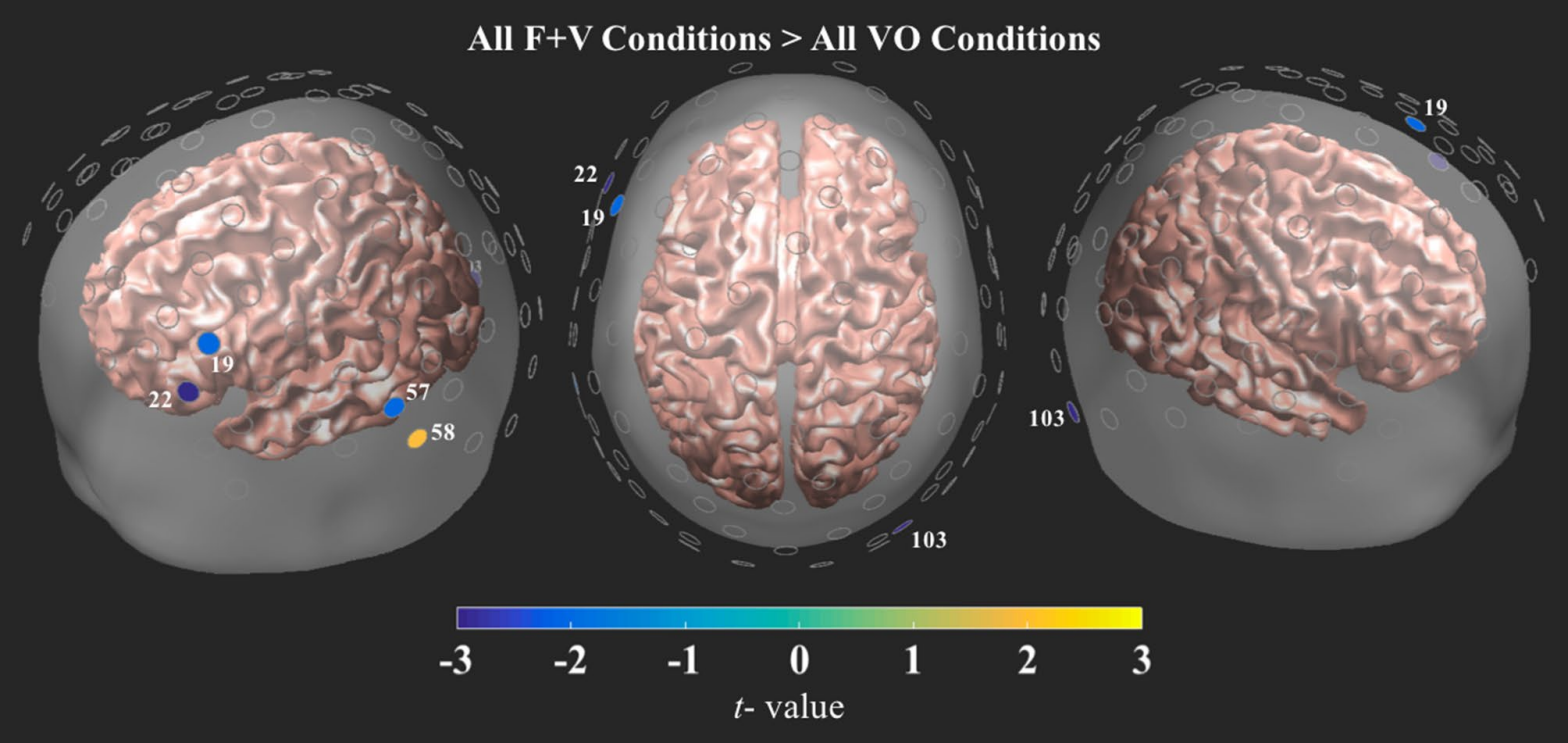
Contrasts for VO conditions. All channels are plotted as gray circles. Channels with significant differences in HbO are shown in yellow or blue, with the associated channel number shown in white. The magnitude and direction of the t-values is represented by the color bar shown on the bottom of the figure. All channels survived FDR correction at $\hbox {q} = .05$.

When contrasted with the Neutral voice condition, both the Angry and Happy voice conditions exhibited decreased activity bilaterally (Fig. 5a,b, see Supplementary Table 4S for a full description of the results). Similar to the activations in the bimodal contrasts, these patterns were not overlapping, with Angry voice showing lower concentrations of HbO in the left IOG and right precentral gyrus (Fig. 5b). The Happy voice condition exhibited decreased HbO in left MTG and posterior areas of the right STG (Fig. 5a). When compared against one another, significant differences were right lateralized, with Angry voice showing greater HbO than the Happy voice condition in the anterior portion of the temporal lobe (Fig. 5c).

#### Mean face and voice, and voice only condition contrast

**Figure 6:**
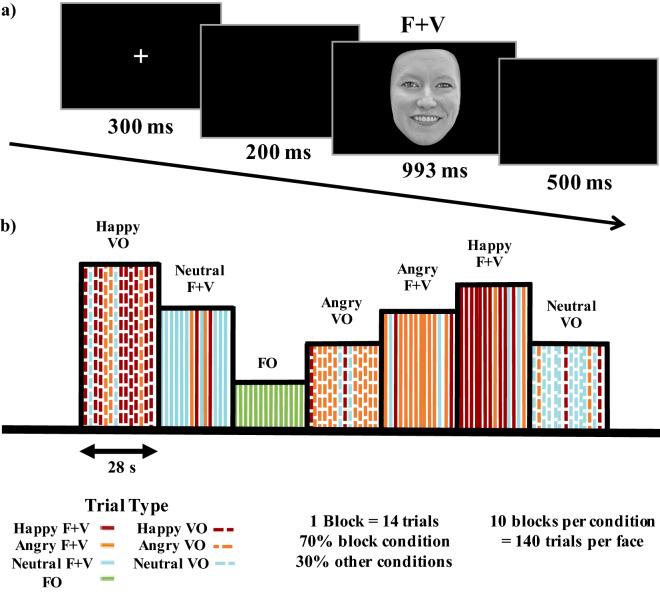
Results from the collapsed F + V > collapsed VO contrasts, collapsed across voice condition. All channels are plotted as gray circles. Channels with significant differences in HbO are shown in yellow or blue, with the associated channel number shown in white. The magnitude and direction of the t-values is represented by the color bar shown on the bottom of the figure. All channels survived FDR correction at $\hbox {q} = .05$.

The mean activity of all F + V conditions was contrasted with the mean activity of all VO conditions. The VO conditions exhibited greater activity in areas associated with language processing in the left hemisphere, with activity appearing in the inferior frontal gyrus and posterior areas of the inferior temporal gyrus (Fig. 6, see Supplementary Table 5S for full details). Additionally, the F + V conditions only exhibited greater HbO activity in the left occipitotemporal junction, this area is related to the identification of visual information.

## Discussion

We evaluated participants’ oxygenated hemoglobin (HbO) levels and 2AFC button-press responses as they were presented with images of affective facial expressions and vocal utterances voiced in a happy, angry, or neutral prosody. This design enabled the comparison of both the unimodal (face or voice presented alone) and bimodal (face and voice (F + V)) components of affect expression. At the behavioral level, voices appeared to aid emotion recognition as subjects displayed the smallest JND values when presented with any bimodal F + V condition compared to when faces were shown alone. Prosody may work to reduce confusion when presented with an ambiguous face, enabling the recipient to react immediately and appropriately. However, this perceptual gain may require more processing as the F + V conditions exhibited significantly longer reaction times than the FO condition. There was partial support of our hypothesis of vocal bias of emotion classification from the Happy F + V condition, but not from the Angry F + V condition. Collectively, these data provide further support for the role of prosody in influencing emotion perception at both the neural and behavioral level.

HbO activity for the bimodal conditions varied by prosody, with the Angry and Happy F + V conditions exhibiting higher HbO levels in bilateral temporoparietal junctions (TPJ) brain regions than in the Neutral F + V condition, and the Angry F + V condition showing greater HbO activity in these areas when compared to the Happy F + V condition. The data showed that the Happy F + V condition exhibited significantly greater HbO activity in these regions when compared to both the Angry and Neutral F + V conditions (Fig.2a,c). While the Happy F + V condition exhibited greater HbO activity in bilateral TPJs than the Neutral F + V condition, differences in TPJ activity between the Happy and Angry F + V conditions only appeared in the right hemisphere. These results are intriguing as the left and right TPJs are associated with different aspects of social communication, with integration of vocal and facial social stimuli occurring in the right TPJ and perspective taking being localized to the left TPJ. This suggests that TPJ activity may be linked to the valence of the prosodic voice, as both conditions contained the same faces, but only differed in the prosody of the voice. The Happy F + V condition may have selectively engaged this area as there was no difference in TPJ activity between the Neutral and Angry F + V conditions (Fig. 2b).

The Happy and Angry F + V conditions also exhibited pronounced differences in patterns of activity in the right hemisphere, with a distinct anterior-posterior division appearing between emotions (Fig. 2a,b). Evidence from aprosodia patients has shown that damage to the right hemisphere may selectively impair prosody related functions as comprehension deficits appear in patients with posterior injuries and difficulties in reproducing prosody manifest with anterior damage^19,20^. Both the anterior and posterior regions of the right hemisphere are essential to multimodal affect perception, as damage to either area may result in deficits in identifying, recognizing, or producing emotional expressions^58^. Additionally, these impairments may be connected to the fundamental role of the right hemisphere in processing paralinguistic information related to a speaker’s age, gender, or emotional state^25,26^. The right hemisphere is thus generally involved in distinguishing and associating vocal and facial information with their relevant conceptual representations. Prosody may therefore be an auditory extension of a speaker’s identity and is thought to have a more pronounced effect on emotion perception than other sensory modalities^59^. The impact of prosody on emotion perception is underscored by the ability of affective voices to bias the perception of emotional facial expressions in the direction of the simultaneously presented prosody (Fig. 2) and this effect is not tied to stimulus valence as both negative and positively valenced stimuli have been shown to bias face perception^31^. One neural substrate potentially related to this perceptual bias may be the posterior superior temporal sulcus (pSTS) of the right hemisphere, as this area exhibits increased activity when presented with emotionally incongruent vocal and facial stimuli^34^. We found that both the Happy and Angry F + V conditions exhibited increased activity in these posterior brain regions consistent with pSTS when compared to the Neutral F + V, suggesting that the pSTS evaluates the congruency of affective audiovisual stimuli and this is not valence specific. Average HbO time course activity for the Happy F + V and Neutral F + V conditions is shown in Supplementary Fig. 1S.

Together, these findings show that activity in the right hemisphere may represent a more general role in speaker identification and that this identification may be mediated by a speaker’s prosody. Results from the current study support and extend this assertion as activity was lateralized to the right hemisphere and seemingly subdivided by valence into two anterior and posterior subdivisions (Fig. 2a,b). A similar functional division has been reported in the stroke literature, where patients sustaining damage to anterior regions in the right hemisphere exhibited deficits in recognizing negatively valenced emotions, but this effect was not seen in patients with posterior damage^60^. These findings complement those of the current study, which found that the Angry F + V condition exhibited increased HbO in the right hemisphere, with activations primarily localized to right frontal and somatomotor areas (Fig. 2b).

In contrast to Anger, a homologous posterior anatomical region has not been reported for positively valenced emotions^60,61^. Rather, happiness appears to be represented in several cortical and subcortical areas, with bilateral activity appearing in somatosensory and posterior association areas^37,38,61^. The Happy F + V condition displayed a similar pattern of posterior activity in right superior parietal and lateral occipital regions, with bilateral activity appearing in the TPJs. Again, while this lateralization may reflect the perceptual weight that vocalizations carry in affect perception, the posterior segregation of activity highlights the involvement of brain areas associated with representing and responding to another individuals’ mental state^37,38^. Additionally, these findings may reflect the automatic mimicry and approach behaviors evoked by happy faces^39^. When compared against one another, the Happy F + V stimuli exhibited greater HbO activity over the right occipital region than the Angry F + V stimuli (Fig. 2c). Most interestingly, the Happy F + V condition also exhibited greater levels of HbO in left dorsolateral prefrontal cortex (DLPFC). These data provide indirect support for the role of the right hemisphere in selectively processing angry facial expressions^9,62^. However, neither hemisphere displays a similar specialization for happy faces^9^. These findings may dually reflect the unilateral specialization of the right hemisphere in processing angry stimuli, as well as the more general, bilateral activity evoked by happy stimuli.

Despite their spatial segregation, the Angry and Happy F + V conditions exhibited overlapping areas of increased HbO brains areas near the STG and the right occipital region (ROR), areas that are essential to the fine discrimination and initial integration of affective vocal and facial cues with their corresponding emotion-specific perceptual-representations^6,63^. Further, these activations were only present when the Angry and Happy F + V conditions were compared to the Neutral F + V condition, but not in direct comparisons between emotion conditions (Fig. 2c), suggesting that while activity in middle STG and ROR may be mediated by emotional valence, the regions do not appear to be emotion specific. This is consistent with findings from fMRI, EEG, MEG, and lesion studies, which have indicated that the ROR and mSTG are sensitive to the physical attributes of affective stimuli but may not encode specific emotions^9,22,25^.

Collectively, these findings emphasize the crucial role of audiovisual integration in affect perception, as faces paired with a happy or angry voice exhibited distinctly different patterns of neural activity and the location of this activity appears to closely correspond to those reported in the face mimicry literature^9,39,42^. In the current study, however, this effect was driven by the prosodic information, since all three F + V conditions utilized the same set of face stimuli and differed only in the prosody they were paired with (angry, happy, neutral). This distinct distribution of activity may be related to the apparent bias in perception that was evident in the behavioral findings, with both the Angry and Happy F + V conditions demonstrating a perceptual shift in subjects’ responses when compared to the face only condition. These findings complement the aprosodia literature by showing that emotion is represented in the brain via a distributed pattern of activity that appears to be socially relevant and valence dependent. Prosody comprehension and expression are vital to emotion perception and the current data indicate that differences in the valence of a speaker’s voice may produce a perceptual bias that corresponds to a distinctive pattern of neural activity that follows the anatomical-functional organization of prosody related areas in the right hemisphere. These data suggest that while the physical qualities of a face play a major role in affect perception this is not entirely independent of the simultaneously presented vocal information. To further parse apart this relationship, activity from the face only and prosody only conditions was compared to the Angry and Happy F + V conditions. Interestingly, when compared to the face only condition, both the Angry F + V and Happy F + V conditions exhibited a similar distributed pattern of increased HbO activity over posterior-anterior areas in the right hemisphere (Fig. 3a,b). The F + V conditions also exhibited greater HbO levels than the prosody only conditions, but this activity was not as diffuse as that witnessed in the face comparisons (Fig. 4a,b). Additional analyses were carried out on the NIRS and behavioral data to check for gender differences. There were no significant differences between the men and women for either dataset.

These findings highlight the inherent interrelatedness of affective vocal and facial expressions. These data also support the notion that affect perception evolves in complexity, with initial processing originating in brain areas that are mutually responsive to all vocal (mSTG) and facial (ROR) displays of emotion. Subsequent integration with conceptually relevant knowledge appears to manifest as a divergence of neural activity, separating the right hemisphere into posterior and anterior subdivisions. Indicating that the processing of affective faces and voices may not be entirely separate. Moreover, these findings underscore the ability of prosody to influence the integration of socially relevant, affective multisensory information using fNIRS. Further disentangling the neural correlates underlying these processes may hold special significance in understanding and treating the deficits in speech and emotion perception exhibited by individuals with autism spectrum disorders or other neurodevelopmental disorders^64,65^.

The current study also has significant limitations that should be acknowledged. The technical problem with behavioral data collection during the fNIRS acquisition prevented us from examining correlations between the behavioral and imaging data. Second, the contribution of subcortical structures to emotion perception cannot be assessed with NIRS due to limitations in depth sensitivity^66^. Third, because we did not acquire electromyogram data the degree of facial mimicry/emotional contagion, if any, could not be assessed in our participants. Finally, the lack of findings for the left dorsolateral frontal region should be taken with caution, as the technical failure of one source optode for some subjects necessitated the interpolation of channels in that region across the entire participant dataset. Additionally, the influence of experimental design must be acknowledged, as these findings stand in stark contrast to those of similar studies investigating affective auditory processing. In particular, Shekhar and colleagues (2019) showed that happy voices caused a more positive response than neutral stimuli in areas of the inferior frontal temporal cortex in two-month old infants using a traditional block design experiment^67^. These data showed an initial increase to happy speech followed by a decrease potentially related to neural habituation. The current study employed a hybrid block design, in which multiple similar stimuli were shown in close repetition. This repetition may have habituated responses to happy stimuli and may account for the decrease in HbO that was seen in the happy VO < neutral VO contrasts. However, it should be noted that these discrepancies may also be related to ongoing neurovascular development as infants have been shown to exhibit inverted hemodynamic responses in bilateral temporal brain regions when compared to healthy adults during a passive auditory listening task^68^.

Despite these limitations, the data presented here provide an initial investigation of the neural correlates underlying emotion perception using a multimodal approach to increase the ecological validity and generalizability of the experiment to other neuroimaging studies. To our knowledge, this is the first fNIRS study to investigate multimodal emotion perception using a high-density optode array^69,70^. The striking anterior-posterior distinction between Angry and Happy F + V conditions in the right hemisphere should be replicated and extended in future studies.

## Methods

### Subjects

Thirty-nine subjects used in the NIRS study were drawn from the undergraduate introductory psychology subject pool volunteers from Colorado State University (See Supplementary Table 6S). The protocol was approved by the Colorado State University Institutional Review Board and all participants provided written informed consent before taking part in the procedures. The experiment was conducted in accordance with all relevant guidelines and regulations. Exclusionary criteria were based on self-report and included past or present neurological or psychiatric diagnosis, history of developmental disability, traumatic brain injury, current tobacco use, neurological disorders, visual acuity of worse than 20/20 without correction, and chronic or current substance abuse within the past three months. Due to a technical error in behavioral data acquisition during the fNIRS experiment, behavioral data from the fNIRS experiment was unable to be analyzed. Rather, a behavioral-only dataset was acquired from a separate set of thirty Colorado State University undergraduate students (15 female) (See Supplementary Table 6S). The groups were compared using a repeated measures ANOVA and there were no significant differences in gender or age, and no subjects participated in both experiments.

### Face stimuli

Face stimuli were taken from the NimStim database^71^. This dataset used untrained actors with natural hair and makeup. One angry and one happy closed-mouth image were selected from a subset of 20 actors (10 men) from the NimStim database. Images were grayscaled and cropped tightly around the face so that no hair, neck or clothing was visible. Two continua were generated, one for each actor, using Psychomorph software^72,73^. Each continuum consisted of two end-point prototype images (angry or happy), which were morphed together in seven steps (two endpoints and 5 morphs, in 12.5% steps) so that the mid-point image would be a 50% combination of each prototype image (for an example continuum see Supplementary Fig. 2S). Individual face templates were created for each end-point image using 182 manually placed points. Faces with closed mouths were selected to facilitate morphing. Face stimuli were presented on an LED monitor at 240 Hz refresh rate located 45 cm in front of the subject. Face stimuli subtended 7.62 degrees of vertical visual angle and 5.72 degrees of horizontal angle.

### Auditory stimuli

Auditory stimuli were obtained from the Montreal Affective Voices database, in which professional actors produced short, nonverbal affective interjections of the vowel /a/, which sounds similar to the /a:/ in ”ah” in spoken English^7^. The current stimuli were chosen because they effectively convey emotion, they are not synthetic, and are free of semantic or linguistic information that may indirectly bias participants responses. Three vocalizations expressed in angry, happy, and neutral prosody were chosen for each actor (1 male and 1 female), resulting in a total of six vocalizations. These stimuli have previously been matched and validated for valence (negative, positive), arousal, and perceived intensity^7^. All vocal stimuli were 993 ms in length.

### Paradigm

**Figure 7:**
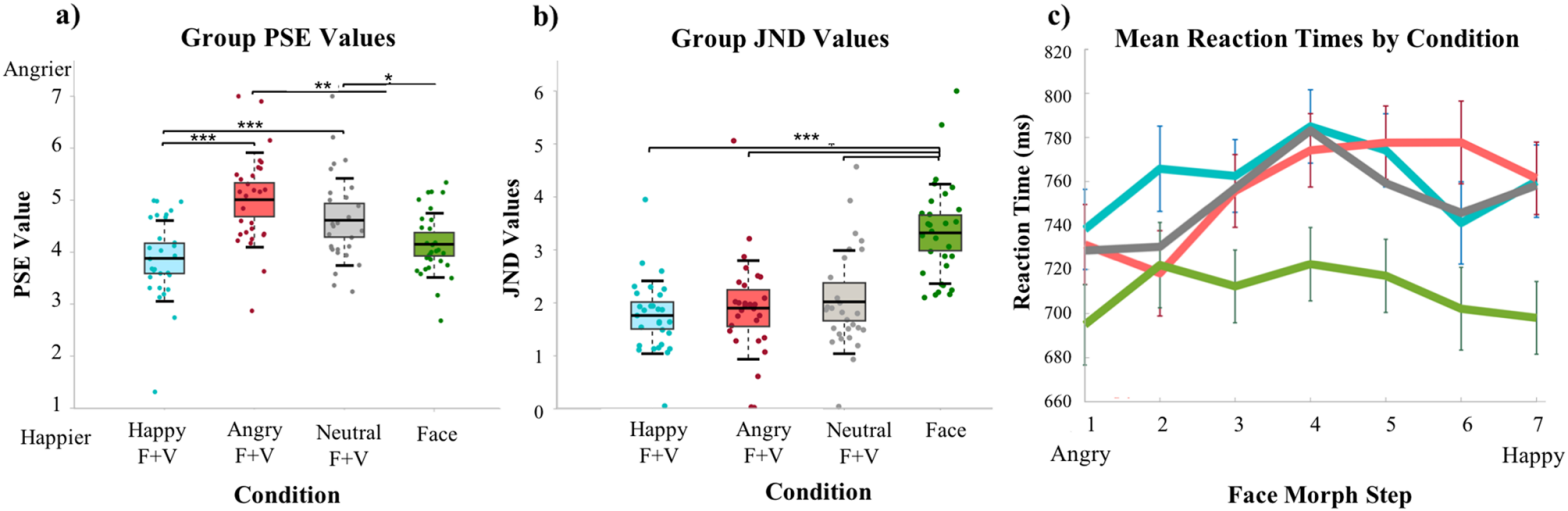
(**a**) Organization and time course for one individual trial. Trial type (face + voice (F + V), voice only (VO), face only (FO)) indicated above stimulus slide. (**b**) Example of hybrid block and trial organization for each trial and block condition.

Participants were presented with three classes of stimuli: face + voice (F + V), voice only (VO), and face only (FO). These stimuli were used to create seven conditions, one for each prosody (happy, angry, neutral) for the F$+$V and VO conditions with one condition for the FO stimuli, see Fig. 7 for an example. Binaural auditory stimulation (70 dB SPL) was delivered via EAR 3a foam insert earphones. Morphed face stimuli were presented alone or simultaneously with auditory stimuli. Each trial began with a white fixation cross on a black background for 300 ms, followed by a 200 ms pause, after which a VO, FO, or F$+$V stimulus was presented for a duration of 993 ms, followed by a blank black screen for 500 ms, for a total trial time of 1993 ms, see Fig. 7a. Subjects were instructed to identify the emotion expressed by the actor for every trial in a two−alternative (“happy” or “not happy”) forced choice (2AFC) procedure using a x−box controller without specific reference to the face or voice. Stimuli were presented using E−Prime 2 Professional (Psychology Software Tools, Inc., United States).

The hybrid block design experiment contained a total of 980 trials (20 actors x 7 conditions x 7 faces on a continuum), with pseudo-random VO, FO, and F + V condition blocks presented as shown in Fig. 7b. Blocks were defined by their stimulus type (FO, F$+$V, VO) and condition (Happy F$+$V, Angry F$+$V, Neutral F$+$V, Happy VO, Angry VO, Neutral VO, FO). All blocks contained 14 trials and were 28 s in duration. Block condition was indicated by the trial type that was in the majority and trials were organized pseudorandomly. For the three F$+$V and VO conditions, 70% of the trials were the same as the block voice condition, and the remaining 30% was divided equally between the two remaining voice conditions. This was done to prevent subjects from easily predicting the emotions within the blocks. Each condition was shown in 10 blocks, for a total of 70 blocks, which were organized in a pseudorandom order. Face and voice gender were matched for all F + V conditions. For the NIRS scan, two resting periods of 2−minute duration were added at the beginning (block 1) and middle (block 37) of the experiment where subjects were instructed to relax, while a central fixation cross was shown on the screen, for a total for 72 blocks. The total duration of the NIRS experiment was 36.5 min, and the total duration for the behavioral experiment was 32.5 min.

### NIRS instrumentation and preprocessing

**Figure 8:**
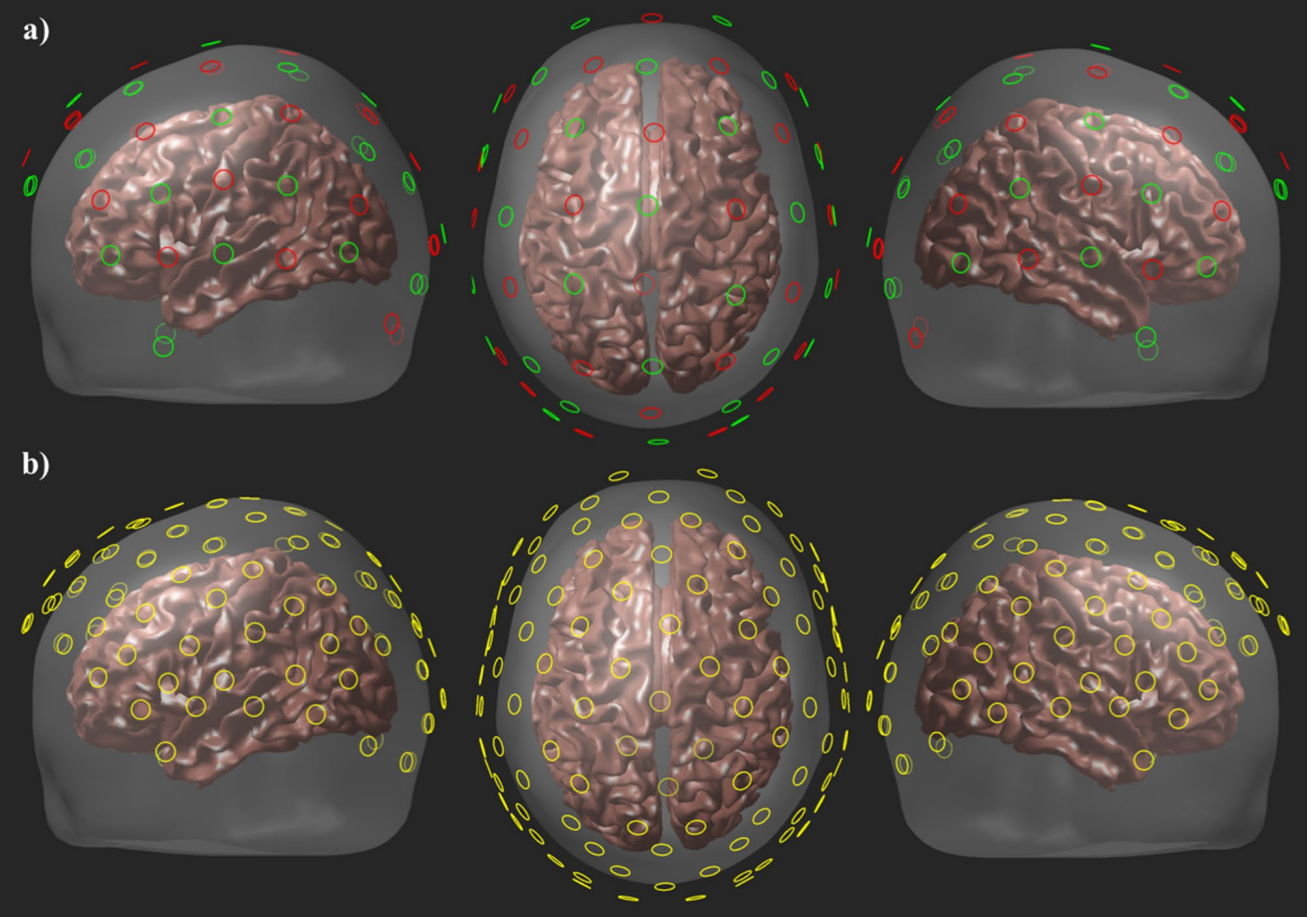
(**a**) Montage showing source (red) and detector (green) positions. (**b**) NIRS channels are located at the halfway point between each source-detector pair and are shown in yellow. NIRS channels were located approximately 3 cm apart, to produce a total of 103 channels.

Optical data were acquired using a continuous wave NIRScoutX (NIRScout; NIRx Medical Technologies, Los Angeles, CA, USA) NIRS system, which can record from up to 32 multiplexed silicon dioxide photodetectors. In our montage, 16 detectors were located over each hemisphere. The optode array contained 28 source positions (light emitting diodes) operating at two wavelengths 760 and 850 nm. Data were acquired at a sampling frequency of 3.92 Hz. Sources and detectors were manually inserted into special NIRS recording caps (Easycap GmbH, Germany) configured in a standard 10−05 International Electrode system manner (Easycap montage M15). This arrangement distributed sources and detectors so that they were located approximately 3 cm apart, to produce a total of 103 channels (See Fig. 8), in an attempt to maximize coverage of the cortical surface and to obtain high−resolution estimates of chromophore concentrations^74^.

Recordings were analyzed in the $spm\_fnirs$ software package for Matlab^75^, where data were cleaned of motion artifact (MARA;^76^) high pass filtered at 0.01 Hz, and temporally smoothed with a 5.0 s moving window to reduce cardiac and respiration noise. Data were compared using nine contrasts (Angry prosody > Neutral prosody, Happy prosody > Neutral prosody, Angry prosody > Neutral prosody, Angry F$+$V > Neutral F$+$V, Happy F$+$V > Neutral F$+$V, Angry F$+$V > Happy F$+$V, all voices > Face only, all face and voices > Face, all F$+$V > all voices). All NIRS channels were first analyzed using a whole−brain approach, by implementing a general linear model design matrix to perform first−level statistics on HbO data. Second−level statistics were performed on all resulting contrasts (p−value .05) to reveal any significant channels. Multiple comparisons were corrected by FDR set at $\hbox {q} = .05$.

### Behavioral data analysis

Reaction times were analyzed using a two-way, 3 (prosody) x 7 (face) repeated measures ANOVA with Greenhouse-Geisser correction for the prosody factor. Significant main effects and interactions were subsequently examined using Bonferroni adjusted Fisher LSD post-hoc tests at $\hbox {alpha} = .05$. Response choices to each face were treated as psychophysical data and were considered as the percent happy responses for each face. The point of subjective equality (PSE, or 50% angry/happy point) and the just-noticeable difference (JND, or 25 + 75% points, divided by 2) were derived from a logistic function fit applied to the 7 face classifications. Each dependent variable was entered into two separate one-way, repeated measures ANOVAs with a single factor of prosody to examine the potential bias of voice prosody on face perception.

## Supplementary information

Supplementary Information.

## Data Availability

The datasets generated during and/or analysed during the current study are available from the corresponding author on reasonable request.
