# Supplementary material for: Prosodic influence in face emotion perception: evidence from functional near-infrared spectroscopy
Source: Sci Rep. 2020 Sep 1;10:14345. doi: 10.1038/s41598-020-71266-6 (PMC7462865; doi:10.1038/s41598-020-71266-6)

Prosodic influence in face emotion perception: evidence from functional near-infrared spectroscopy

Katherine M. Becker, Donald C. Rojas

Colorado State University
Department of Psychology
Campus Delivery 1876
Fort Collins, 80523, USA

Supplementary Tables

Table 1S: Significant channels and anatomical locations for all Face+Voice (F+V) contrasts. All tables show NIRS channel locations in MNI coordinates with source-detector pairs given according to their 10/05 EEG electrode positions. Sources are shown on the left of each electrode pair. All channels significant at p < .05, corrected. Contrasts are bolded.

Brain Area	NIRS Channel	S-D Pair	Channel Location x	Channel Location y	Channel Location z	*t* value
Happy F+V > Neutral F+V						
Bilateral supplementary motor area	5	FCz – Cz	0.4	9.1	94.5	2.72
L Supplemental motor area	6	FCz – FC1	-16.8	26.7	84.3	2.50
L Superior frontal gyrus	12	C1 – Cz	-17.9	-9.6	95.0	2.37
L Superior postcentral gyrus	15	C1 – CP1	-35.8	-28.6	90.5	2.14
R Middle superior temporal gyrus	49	C6 – FC6	81.5	3.6	26.8	2.86
L Occipitotemporal junction	57	TP7 – CP5	-82.2	-46.3	12.0	2.09
L Temporoparietal junction	62	CP3 – P3	-58.3	-62.9	60.8	2.74
R Superior parietal lobule	66	CPz – CP2	19.4	-47.2	95.1	2.33
L Superior occipital gyrus	74	P1 – PO1	-32.6	-90.7	56.3	2.21
R Posterior superior temporal sulcus	86	CP4 – CP6	75.0	-46.4	48.4	2.55
R Temporoparietal junction	87	CP4 – P4	61.1	-62.6	61.1	2.48
R Occipitotemporal junction	89	TP8 – CP6	84.4	-45.8	12.0	2.34
R Posterior parietal cortex	92	P2 – CP2	35.2	-63.8	83.7	2.91
R Superior occipital gyrus	98	P6 – PO2	52.3	-88.4	32.2	3.14
R Middle occipital gyrus	100	PO4 – PO6	36.9	-110.6	-2.2	2.24
Angry F+V > Neutral F+V						
R Superior frontal gyrus	37	C2 – FC2	36.2	8.4	83.6	3.35
R Superior precentral gyrus	38	C2 – C4	52.4	-10.3	76.0	2.26
R Inferior postcentral gyrus	48	C6 – C4	75.3	-11.8	46.4	2.72
R Middle superior temporal gyrus	49	C6 – FC6	81.5	3.6	26.8	2.24
R Supramarginal gyrus	84	CP4 – C4	66.9	-28.8	64.6	2.25
Angry F+V > Happy F+V						
L Superior postcentral gyrus	15	C1 – CP1	-35.8	-28.6	90.5	-2.17
L Dorsolateral prefrontal cortex	16	FC3 – FC1	-47.1	24.4	67.8	-2.39
L Inferior precentral gyrus	18	FC3 – C3	-62.8	5.5	60.0	-3.15
R Inferior postcentral gyrus	48	C6 – C4	75.3	-11.8	46.4	2.23
R Superior occipital gyrus	98	P6 – PO2	52.3	-88.4	32.2	-2.13

Table 2S: Significant channels and anatomical locations for all Face Only (FO) contrasts.

Brain Area	NIRS Channel	S-D Pair	Channel Location			*t* value
			x	y	z	
Happy F+V > FO						
Bilateral supplementary motor area	5	FCz – Cz	0.4	9.1	94.5	2.34
L Supplemental motor area	6	FCz – FC1	-16.8	26.7	84.3	2.41
R Superior parietal lobule	66	CPz – CP2	19.4	-47.2	95.1	2.47
R Temporoparietal junction	87	CP4 – P4	61.1	-62.6	61.1	2.15
R Posterior parietal cortex	92	P2 – CP2	35.2	-63.8	83.7	2.70
R Superior occipital gyrus	98	P6 – PO2	52.3	-88.4	32.2	3.45
Angry F+V > FO						
L Dorsolateral prefrontal cortex	16	FC3 – FC1	-47.1	24.4	67.8	-2.08
R Superior frontal gyrus	37	C2 – FC2	36.2	8.4	83.6	2.69
R Inferior postcentral gyrus	48	C6 – C4	75.3	-11.8	46.4	2.84
R Superior parietal lobule	66	CPz – CP2	19.4	-47.2	95.1	2.21
R Supramarginal gyrus	84	CP4 – C4	66.9	-28.8	64.6	2.25
R Superior parietal lobule	85	CP4 – CP6	52.5	-46.9	78.1	2.67

Table 3S: Significant channels and anatomical locations for all Voice Only (VO) contrasts.

Brain Area	NIRS Channel	S-D Pair	Channel Location			*t* value
			x	y	z	
Happy F+V > Happy VO						
R Supramarginal gyrus	86	CP4 – P4	75.0	-46.4	48.4	2.55
R Superior occipital gyrus	98	P6 – PO2	52.3	-88.4	32.2	2.37
Angry F+V > Angry VO						
L Dorsolateral prefrontal gyrus	17	FC5 – FC3	-55.2	37.9	48.9	-2.17
R Superior frontal gyrus	37	C2 – FC2	36.2	8.4	83.6	2.75
R Precentral gyrus	38	C2 – C4	52.4	-10.3	76.0	2.12
R Anterior middle temporal gyrus	52	FT8 – FC6	80.7	17.7	6.6	-2.42
R Inferior anterior temporal gyrus	55	FT10 – F10	83.0	14.9	-30.9	-2.04
L Inferior occipital gyrus	76	PO9 – PO5	-48.9	-103.1	-24.3	2.34

Table 4S: Significant channels and anatomical locations for all Voice Only (VO) contrasts.

Brain Area	NIRS Channel	S-D Pair	Channel Location			*t* value
			x	y	z	
Happy VO > Neutral VO						
L Middle temporal gyrus	26	C5 – T7	-70.0	-19.7	-17.3	-2.41
R Supramarginal gyrus	86	CP4 – P4	67.0	-50.3	23.7	-2.27
Angry VO > Neutral VO						
R Precentral gyrus	38	C2 – C4	59.0	-11.3	51.7	-3.14
L Inferior occipital gyrus	76	PO9 – PO5	-33.7	-89.3	-26.3	-2.35
Angry VO > Happy VO						
R Inferior frontal gyrus	45	F6 – F4	53.0	44.7	-10.0	2.11
R Anterior middle temporal gyrus	52	FT8 – FC6	63.0	6.0	-23.0	2.58

Table 5S: Significant channels and anatomical locations for the Voice Only (VO) contrast, collapsed across happy, angry, and neutral Face+Voice conditions (HAN), and VO conditions (HAN Voice).

Contrast	NIRS Channel	S-D Pair	Channel Location			*t* value
			x	y	z	
All F+V conditions > All VO conditions						
L Inferior frontal gyrus	19	FT7 –T7	-68.7	20.7	40.0	-2.11
L Inferior frontal gyrus	22	CPz – CP2	-70.8	33.3	20.7	-3.18
L Inferior temporal gyrus	57	TP7 – CP5	-82.2	-46.3	11.9	-2.13
L Occipitotemporal junction	58	TP7 – P9	-78.6	-59.7	-4.8	2.03
R Inferior occipital gyrus	103	O10 – O2	49.4	-103.6	-24.4	-3.85

Table 6S: Subject demographics.

**Dataset**	***n***	**Age**	**Age Range**	**Gender**	
		M(SD)		M	F
NIRS	39	20.37(1.19)	19-24	17	22
Behavioral	30	21.03(3.35)	19-31	15	15

Table 7S: Mean questionnaire scores concerning drug and alcohol use, and general physical and mental health.

		NIRS		Behavioral	
Scale	Variable	*M*	*SD*	*M*	*SD*
DUKE					
	Physical health	83.33	14.43	83.45	13.17
	Mental health	82.86	11.75	82.50	15.78
	Social health	87.38	13.26	84.83	15.26
	General health	84.49	9.46	83.57	9.99
	Perceived health	85.71	22.86	83.93	23.78
	Self-esteem	91.19	10.87	87.50	11.10
	Anxiety	20.24	13.54	20.69	14.88
	Depression	19.05	16.79	22.50	17.56
	Anxiety-depression	19.22	13.62	19.39	14.53
	Pain	23.81	27.58	17.24	24.19
	Disability	3.57	13.03	3.45	12.89
DAST-10					
	Drug abuse	1.37	0.76	1.74	1.44
AUDIT					
	Alcohol use	3.89	2.78	3.84	2.85

Table 8S: Means and standard deviations for the Point of Subjective Equality (PSE) and Just Noticeable Difference (JND) values for each Face+Voice (F+V) condition and the Face only condition.

Condition	PSE	JND
	M(*SD*)	*M*(*SD*)
Happy F+V	3.78(0.95)	1.77(0.69)
Angry F+V	4.87(1.14)	2.04(1.20)
Neutral F+V	4.70(0.97)	2.16(1.20)
Face Only	4.15(0.63)	3.32(0.94)

Figure 1S: Average HbO time course for the Happy F+V and Neutral F+V conditions for a single channel in one subject.

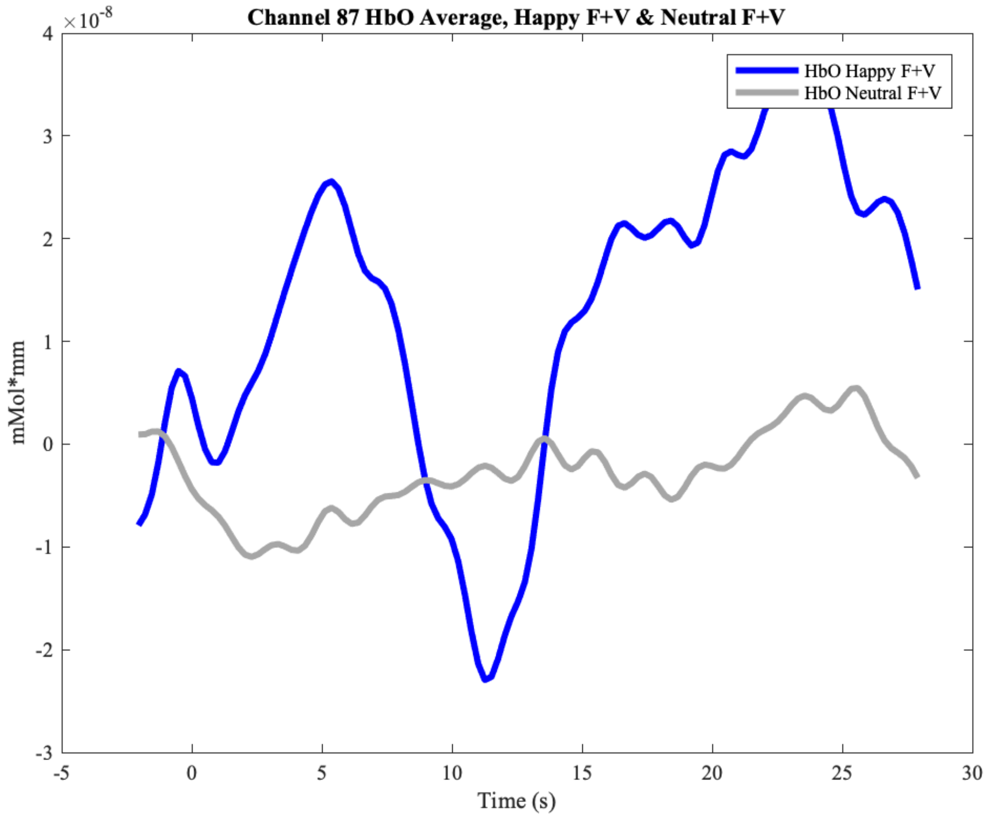

Figure 2S: Example of a single morphed face continuum generated from one happy and one angry face.

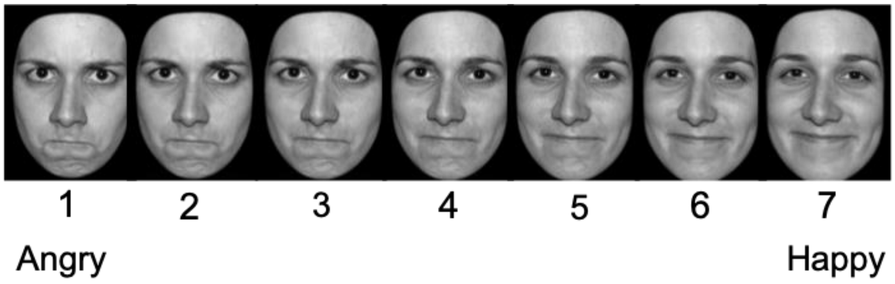

Supplement: Supplementary file 1 — Supplementary Information. [file 41598_2020_71266_MOESM1_ESM.pdf]
